# Regulatory T cells and their role in rheumatic diseases: a potential target for novel therapeutic development

**DOI:** 10.1186/1546-0096-6-20

**Published:** 2008-12-01

**Authors:** Diana Milojevic, Khoa D Nguyen, Diane Wara, Elizabeth D Mellins

**Affiliations:** 1 Department of Pediatrics, UCSF, San Francisco, CA 94143, USA; 2 Department of Pediatrics, Stanford University, Stanford, CA 94305, USA

## Abstract

Regulatory T cells have an important role in limiting immune reactions and are essential regulators of self-tolerance. Among them, CD4+CD25^high ^regulatory T cells are the best-described subset. In this article, we summarize current knowledge on the phenotype, function, and development of CD4+CD25^high ^regulatory T cells. We also review the literature on the role of these T cells in rheumatic diseases and discuss the potential for their use in immunotherapy.

## Introduction

**Figure 1:**
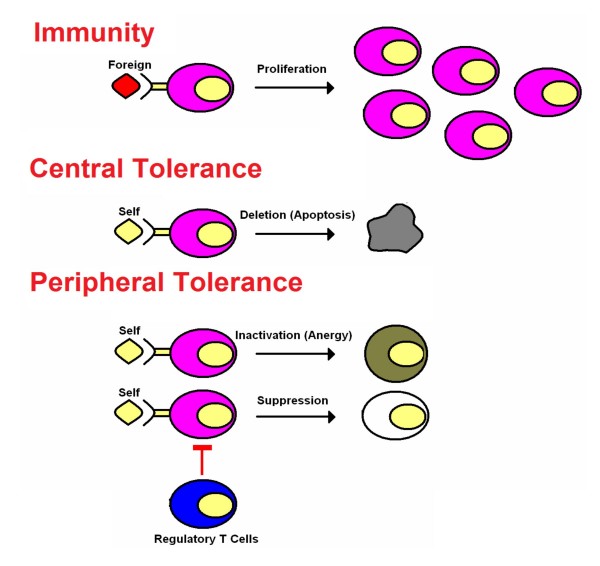
Mechanisms of immune tolerance.

Tolerance to "self" is a major immune regulatory mechanism that protects the body's own tissues from immune-mediated damages and restricts active immune responses to those against microbial invaders (Figure 1). A classical type of tolerance, called central tolerance, is the mechanism by which "forbidden clones" of lymphocytes that recognize self antigens are eliminated in the thymus during normal lymphocyte development [1-3]. However, some lymphocyte clones with specificities for self antigens are found in animals and humans without autoimmunity [4-8]. In addition, autoimmunity can develop in the absence of defects in central tolerance. These findings initially led to the hypothesis that peripheral tolerancemust prevent auto-aggression by self-reactive T cells that escape thymic deletion. In the 1970s and 1980s, work on peripheral tolerance focused on characterization of specific suppressor T cells, the presumed regulators of immune responses in the periphery [9]. However, attempts to define and isolate suppressor T cells led to conflicting results, disappointment, and near abandonment of the field. With the development of new technologies in the 1990s, compelling evidence was put forward to support the existence of cellular subsets that possess immunosuppressive activities, this time under the name regulatory T cells[10].

### Types of regulatory T cells

**Figure 2:**
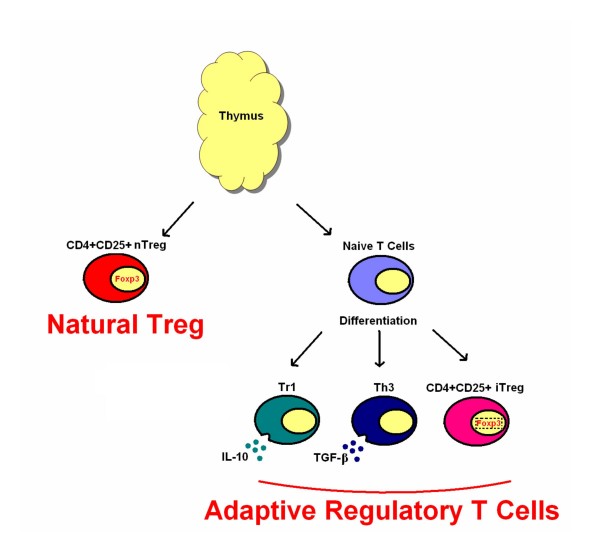
Different subsets of regulatory T cells.

There are various types of regulatory T cells, including TCRαβ+CD4+, TCRαβ+CD8+, TCRαβ+CD4-CD8-, and TCRγι+ T cells. The majority of recent research has focused on TCRαβ+CD4+ regulatory T cells, of which there are several subtypes with distinct surface phenotypes, cytokine production profiles and mechanisms of immune suppression. Among the subtypes, T cells produced in the thymus and delivered to the periphery as a long-lived lineage of self-antigen-specific lymphocytes are called natural CD4+CD25^high ^regulatory T cells (nTreg). In contrast+, CD4+ T cells that are recruited from circulating lymphocytes and acquire regulatory properties under particular conditions of stimulation are called adaptive Tcells(Figure 2). Two types of adaptive CD4+ regulatory T cells are type 1 regulatory T cells (Tr1) and T helper 3 regulatory cells (Th3). Suppressive effects of Tr1 and Th3 cells are dependent on the production of inhibitory cytokines, IL-10 and TGF-β, respectively [11-18]. A third type of adaptive regulatory T cell is the CD4+CD25^high ^T cell induced in the periphery; these are termed induced regulatory T cells (iTreg). iTreg have similar properties to thymus-generated nTreg. Both cell types are anergic and do not proliferate upon TCR stimulation. Both cell types can inhibit proliferation of CD4+CD25- T cells in a dose dependent manner. Despite their characteristic anergy, CD4+CD25^high ^regulatory T cells cultured with anti-CD3 antibodies (for TCR stimulation) and excess IL-2 (a T cell growth factor), can proliferate and still retain their suppressive activities. CD4+CD25^high ^regulatory T cells (nTreg and iTreg) are the subject of this review.

### Development of CD4+CD25^high ^regulatory T cells

NTreg arise during normal lymphocyte ontogeny in the thymus [18,19], and this is thought to be the exclusive site of nTreg development in children [20]. NTreg represent 5–10% of CD4+CD8- thymocytes in humans, mice, and rats. It seems likely that nTreg are positively selected through high-affinity recognition of self peptides presented by thymic stromal cells. This event, possibly together with signals from thymic dendritic cells, stimulates production of anti-apoptotic molecules to protect against negative selection. Recent data also indicate that CD4+CD25^high ^regulatory T cells have a reciprocal developmental relationship in with Th17 cells, inflammatory T helper cells that produce IL-17 [21].

Many aspects of nTreg development in the thymus, such as their site of development, their interaction with thymic epithelial cells, and their selection are still poorly understood [22,23]. Despite these uncertainties, it is clear that the transcription factor forkhead box P3 (Foxp3) plays a major role in the ontogeny and function of nTreg [23-29]. FoxP3 is preferentially and stably expressed in peripheral nTreg, even after proliferation [23,27]. However, the signals that induce the stable up-regulation of Foxp3 and committed regulatory function in nTreg are not known. Furthermore, recent research shows that much of the nTreg transcriptional signature is not ascribable to Foxp3. It seems that a complex regulatory mechanism upstream of Foxp3 determines nTreg lineage and is distinct from elements downstream of Foxp3 that are essential for the cell's regulatory properties [30]. After their thymic selection, nTreg populate peripheral tissues. They are believed to be long-lived and may repeatedly proliferate in the periphery upon encountering specific self antigens [31-33]. However, their potential for continuous cell division is limited, which is thought to be associated with their diminished telomerase activity compared to CD4+CD25- T cells [34,35].

The total number of CD4+CD25^high ^regulatory T cells in human peripheral blood increases with age, despite thymic involution [36]. The likely explanation is the thymus-independent generation of CD4+CD25^high ^iTreg. Several lines of evidences have suggested that induction of iTreg requires FoxP3. When a Foxp3 gene is transduced into CD4+CD25- T cells, these cells acquire CD25 surface expression and other phenotypic characteristics of nTreg. These transduced CD4+CD25^high ^iTreg are able to inhibit proliferation and cytokine production in the effector T cells and the development of some experimental autoimmune diseases in animals [37]. Murine and human studies show that several cytokines are also required for generation of extra-thymic CD4+CD25^high ^iTreg. Essential stimuli include TGF-β [17,38-41], IFN-γ [42], anti-CD3/CD28 antibodies or antigen specific stimulation [43,44], IL-4/IL-13 [45,46], and thrombospondin-CD47 interaction [46]. Murine studies also show that tolerogenic conditions and homeostatic proliferation during lymphopenia induce the development of CD4+CD25^high ^Foxp3+ iTreg *in vivo *[47-51].

### Phenotype of CD4+CD25^high ^regulatory T cells

**Figure 3:**
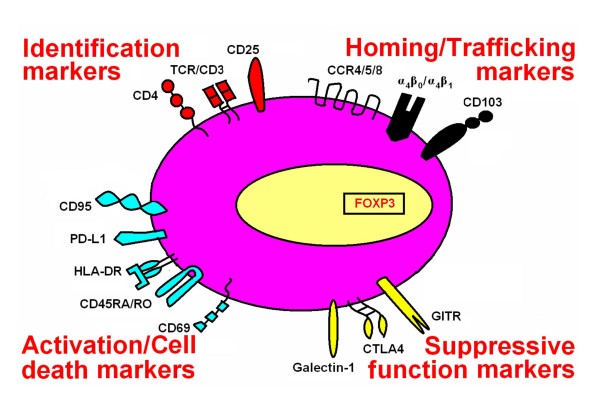
Surface markers associated with CD4+CD25high regulatory T cells.

No specific marker for CD4+CD25^high ^regulatory T cells is yet known (Figure 3). Foxp3 has been considered the most reliable marker [23], but is intracellular and cannot be used for isolation or *in vivo *tracking of CD4+CD25^high ^regulatory T cells. In addition, activation of CD4+CD25- T cells can transiently up-regulate FoxP3 expression in human cells, although this is not the case in mice [41,52,53]. Hence, FoxP3 alone may not be a specific marker for human CD4+CD25^high ^regulatory T cells [53].

Another molecule associated with CD4+CD25^high ^regulatory T cells is CD25, the α chain of the IL-2 receptor, Both nTreg and iTreg constitutively express CD25 and suppressive activity is optimal in CD4+ T cells expressing the highest levels of CD25 (approximately 2–4% of human peripheral blood CD4+ T cells). However, CD25 by itself has limitations as a marker for CD4+CD25^high ^regulatory T cells, as it is also up-regulated in activated effector T cells. The recent discovery of low expression of CD127 (IL-7 receptor α) on CD4+CD25^high ^regulatory T cells provides further delineation of this population [54-56]. However, some regulatory CD4+ T cells that are Foxp3+CD127^low ^express little-to-no CD25 [56].

Several other molecules associated with CD4+CD25^high ^regulatory T cells have been descsribed. In humans, these cells constitutively express intracellular cytotoxic T-lymphocyte antigen 4 (CTLA-4) and glucocorticoid-induced tumor-necrosis-factor-receptor-related protein (GITR). Upon activation, they also express membrane-bound TGF-β and HLA-DR [57]. Other surface markers reportedly expressed on human CD4+CD25^high ^regulatory T cells include CD69, CD45RA/CD45RO, CD134 (OX40), CD95, and programmed cell death-ligand 1 (PD-L1). CD4+CD25^high ^regulatory T cells also express chemokine receptors to direct their migration to different tissues. Current data suggest that signals from various chemokines and integrin ligands determine which membrane chemokine receptors and integrins are expressed on CD4+CD25^high ^regulatory T cells. Similar to effector T cells, CD62L (also known as L-selectin) and CCR7 are important lymph node homing molecules for CD4+CD25^high ^regulatory T cells [58]. The majority of CD4+CD25^high ^regulatory T cells express CCR4 and CCR8 [59], but other chemokine receptors and integrin molecules, such CD103, are also present. The expression level of integrins dictates the direction of cell migration. For example, CD4+CD25^high ^CD103- regulatory T cells preferentially migrate to lymph nodes, whereas CD4+CD25^high ^CD103+ regulatory T cells efficiently migrate into inflammatory sites [58]. Most human CD4+CD25^high ^regulatory T cells are believed to be in a late stage of differentiation. This notion is supported by their expression of activation/memory markers, as indicated above [60].

The absence of specific markers makes it difficult to isolate pure populations of CD4+CD25^high ^regulatory T cells, to further characterize their phenotype. At least a small number of non-regulatory activated effector T cells usually contaminate isolated CD4+CD25^high ^regulatory T cells, due to the overlapping expression of CD25. Thus, strategies to expand CD4+CD25^high ^regulatory T cells for higher yield and purity have been sought. Use of IL-2, a T cell growth factor that induces proliferation of CD4+CD25^high ^regulatory T cells *in vitro*, was considered. However, IL-2 also favors the expansion of non-regulatory effector T cells. Another candidate is the immunosuppressive drug rapamycin (sirolimus), used for the prevention of organ transplant rejection as well as resistant graft versus host disease (GVHD) [61-63]. Human peripheral blood CD4+CD25^high ^regulatory T cells cultured in the presence of rapamycin survive and vigorously expand for at least 3 weeks, while effector T cells are inhibited from proliferation. This phenomenon is thought to result from differential intracellular signaling in CD4+CD25^high ^regulatory T cells compared to CD4+CD25- effector T cells in response to rapamycin, which blocks progression from G1 into S phase in activated effectors [64]. The rapamycin-expanded CD4+CD25^high ^regulatory T cells are suppressive and have the same phenotype as freshly isolated blood CD4+CD25^high ^regulatory T cells. Thus, *in vitro *rapamycin may allow the generation of highly efficient CD4+CD25^high ^regulatory T cells and better characterization of their functions for potential clinical use [65,66].

### CD4+CD25^high ^regulatory T cell function

A key characteristic of CD4+CD25^high ^regulatory T cells is their *in vitro *anergy. In contrast to CD4+CD25- T cells, which proliferate upon receiving T cell receptor (TCR) stimulation, CD4+CD25^high ^regulatory T cells are unresponsive to this proliferative signal and do not produce IL-2. However, CD4+CD25^high ^regulatory T cells cultured with anti-CD3 antibodies for TCR stimulation and excessexogenous IL-2 overcome anergy and proliferate; blocking IL-2 inhibits this phenomemon [67]. The anergic state of CD4+CD25^high ^regulatory T cells can also be overcome by anti-CD28 costimulation or interaction with mature dendritic cells [68-70]. Interestingly, recent studies suggest that CD4+CD25^high ^regulatory T cells are not anergic *in vivo*, but have a high turnover rate [71,72].

The second cardinal feature of CD4+CD25^high ^regulatory T cells is their ability to suppress immune responses [72,73]. Suppression occurs when CD4+CD25^high ^regulatory T cells are activated with antigens recognized by their specific TCR, but can be maintained without further TCR stimulation [74]. Thus, suppressive activity is antigen-nonspecific. However, CD4+CD25^high ^regulatory T cells that share the same antigenic specificity with effector cells are more suppressive. Similarly, allogeneic CD4+CD25^high ^regulatory T cells are suppressive, but autologous CD4+CD25^high ^regulatory T cells are more potent suppressors. Some studies suggest that CD4+CD25^high ^regulatory T cells inhibit proliferation of effector CD4+CD25- T cells and CD8+ T cells by arresting the proliferation of these cells at G1-S interphase of the cell cycle [75]. Interestingly, the addition of exogenous IL-2 does not overcome the suppression, suggesting unresponsiveness at the level of the IL-2 receptor [72]

Contact-dependent suppression by CD4+CD25^high ^regulatory T cells has been reported to occur via CTLA-4 signaling: CTLA-4 blockade leads to diminished suppression of effector T cell proliferation by CD4+CD25^high ^regulatory T cells [76,77]. Recent studies have suggested that multiple CTLA-4 associated pathways could mediate suppression by CD4+CD25^high ^regulatory T cells. Preferential engagement of CTLA-4, instead of CD28, with CD80/CD86 may provide a negative proliferative signal [78]. Alternatively, CTLA-4 on CD4+CD25^high ^regulatory T cells may signal dendritic cells to produce the immunosuppressive cytokines, IL-10 and TGF-β [79]. In a novel mechanism, suggested by results of Fallarino et al., CTLA-4 signals dendritic cells to produce high levels of the enzyme indoleamine, which in turn breaks down tryptophan, an amino acid important for T cell proliferation [80], and consequentially inhibits the proliferation of effector T cells.

While the main targets of suppression by CD4+CD25^high ^regulatory T cells are innate and adaptive immune cells [81], these regulatory T cells also participate in immune responses against infectious agents [82], malignant cells [83], and allogeneic organ and stem-cell grafts [84]. Although CD4+CD25^high ^regulatory T cells regulate both Th1 and Th2 immune responses, Th2 cells may partially escape this suppressive activity via their ability to respond to growth factors other than IL-2, such as IL-4, IL-7, and IL-9 [85]. In contrast, the proliferation of Th1 cells is only restored by the administration of IL-15 [85]. In mice, the depletion of CD4+CD25^high ^regulatory T cells prevents antigen-induced Th2 differentiation by increasing the differentiation of Th1 cells [86,87]. Under appropriate conditions, CD4+CD25^high ^regulatory T cells are able to confer suppressive capacity on CD4+CD25- T cells, converting them to either Th3 or Tr1 cells [88,89].

### CD4+CD25^high ^regulatory T cells and autoimmunity

Several autoimmune disorders have been linked to physical and genetic alterations in thymus that disrupt the development of nTreg. Thymectomized neonatal mice are deficient in CD4+CD25^high ^regulatory T cells and develop multi-organ autoimmune disease, which can be overcome by the adoptive transfer of CD25+ thymocytes from normal mice [90,91]. Children with thymic hypoplasia as a result of the 22.q2 deletion syndrome display impaired CD4+CD25^high ^regulatory T cell generation and have an increased risk of developing an autoimmune disorder [92]. Mutations in Foxp3 result in the scurfy phenotype in mice. Foxp3 mutant "scurfy" mice and Foxp3-null mice lack CD4+CD25^high ^regulatory T cells and die of a lymphoproliferative-wasting disease, likely due to uncontrolled expansion of effector T lymphocytes. Adoptive transfer of CD4+CD25^high ^regulatory T cells into neonatal Foxp3-null or scurfy mice protects them temporarily from disease [92,93].

Human patients with Foxp3 gene mutations develop IPEX syndrome, a potentially fatal disorder, characterized by immune dysregulation, polyendocrinopathy, and enteropathy (Table 1) [94-96]. IPEX CD4+CD25^high ^regulatory T cells are less suppressive, although their surface phenotype and levels in peripheral blood remain normal [97]. Consequently, it is suggested that functional insufficiency rather than defective differentiation of CD4+CD25^high ^regulatory T cells may occur in these patients. Allogeneic bone marrow transplantation in IPEX subjects is effective in correcting Foxp3 associated dysfunctions [98], and clinical recovery accompanies regeneration of functionally competent CD4+CD25^high ^regulatory T cells [99].

**Table 1:** Characteristics of IPEX Syndrome ^#§^.

**Organ system**	Manifestations*
Endocrine	Insulin dependent diabetes mellitus Thyroid dysfunction Parathyroid hormone resistance

Gastrointestinal	Autoimmune enteropathy Diarrhea Villous atrophy, failure to thrive

Skin	Eczema Ichthyosiform dermatitis Exfoliative dermatitis

Infectious disease	Exaggerated response to viral infections Frequent infections

Immune dysregulation	Increased IgE, intermittent eosinophilia Skewing of T lymphocytes to Th2 phenotype Hemolytic anemia Immune thrombocytopenia Coagulopathy

^# ^Alternative names: XLAAD (X-linked autoimmunity allergic dysregulation syndrome); insulin dependent diabetes mellitus-secretory diarrhea syndrome; XPID (Polyendocrinopathy, immune dysfunction, diarrhea, X-linked)

^§ ^Treatment: immunosuppression (Cyclosporine A), allogeneic bone marrow transplantation

* Manifestations of the disease are highly variable

In addition to IPEX, many more common polygenic autoimmune disorders, including multiple sclerosis, type 1 diabetes, are hypothesized to have abnormalities in CD4+CD25^high ^regulatory T cell function [100-105]. Below, we consider this hypothesis and discuss findings from studies of these cells in rheumatic diseases. Across the spectrum of autoimmune diseases, it is not yet clear whether changes in these cells are primary or secondary to disease.

### CD4+CD25^high ^regulatory T cells in rheumatic diseases

In rheumatic diseases, most studies have focused on CD4+CD25^high ^regulatory T cells, while the roles of other regulatory T cell types remain unclear (Table 2). Early attempts to characterize CD4+CD25^high ^regulatory T cells were flawed due to use of high surface expression of CD25 as the single cell marker and the resulting inclusion of variable numbers of activated T effector cells over the course of disease. In addition, levels and/or activity of CD4+CD25^high ^regulatory T cells are influenced by different immunosuppressive treatments. Therefore, future studies that employ a better combination of markers (e.g. CD4, CD25, and CD127) and consider medication status and disease severity in the analysis will be important. Nonetheless, current studies of CD4+CD25^high ^regulatory T cells in rheumatic diseases provide the scientific foundation for further research.

**Table 2:** CD4+CD25^high ^regulatory T cells in rheumatic diseases.

**Disease**	**Abnormalities associated with regulatory T cells**	**Authors**
**JIA**	1) ↓ numbers in extended oligoarticular JIA. 2) HSP epitopes induce ↑ numbers in synovial fluid. 3) ASCT induces restoration of normal numbers and immune tolerance. 4) Regulatory T cells from inflamed joints express CD27.	1) DeKleer et al., 2004 (106). 2) Massa et al., 2007 (109). 3) DeKleer et al., 2006 (110). 4) Ruprecht et al., 2005 (111).

**RA**	1) No change in numbers in peripheral blood, ↑ numbers in synovial fluid. 2) Anti-TNF-α treatment does not induce changes in numbers and function*. 3) Anti-TNF-α treatment induces ↑ in numbers and function *. 4) Synovial T cells are more resistant to suppression. 5) ↑ numbers of CCR5+ CXCR4+ regulatory T cells in synovial fluid. 6) Imbalance between IFN-γ producing cells and regulatory T cell numbers.	1) Cao et al., 2003 (108), Mottonen et al., 2005 (119). 2) Dombrecht et al., 2006 (115). 3) Ehrenstein et al., 2004 (117). 4) van Amelsfort et al., 2004 (116). 5) Jiao et al., 2007 (120). 6) Behrens et al., 2007 (121).

**SLE**	1) ↓ numbers during active disease*. 2) No change in numbers during clinical remission. 3) ↑ numbers*. 4) Treatment does not induce changes in numbers*. 5) Corticosteroid treatment induces ↑ numbers*. 6) IFN-α producing cells block suppressive function. 7) Positive correlation between numbers and disease severity*. 8) Inverse correlation between numbers and disease severity*. 9) Reversible functional defect in active disease.	1) Liu et al., 2004 (126); Mellor-Pita et al., 2006 (127). 2) Crispin et al., 2004 (128). 3) Azab et al., 2008 (129). 4) Cepika et al, 2007 (131) 5) Valencia et al., 2007 (132). 6) Yan et al., 2008 (134). 7) Mellor-Pita et al., 2006 (127). 8) Lin et al., 2007 (130). 9) Barath et al., 2007 (135).

**Spondylo-arthropathy**	1) No change in numbers and function.	1) Cao et al., 2003 (108).

**Kawasaki disease**	1) ↓ numbers in active disease; normalized numbers in defervescense.	1) Furuno et al., 2004 (138).

**Sjogren's syndrome**	1) ↑ numbers*. 2) ↓ numbers*.	1) Gottenberg et al., 2005 (136). 2) Li et al., 2007 (137).

**Sarcoidosis**	1) ↑ numbers; insufficient inhibition of TNF-α production. 2) ↓ regulatory T cell-associated genes in broncho-aveolar fluid T cells.	1) Miyara et al., 2003 (139). 2) Idali et al., 2008 (140).

* Conflicting results.

### Juvenile idiopathic arthritis (JIA)

Research on CD4+CD25^high ^regulatory T cells in juvenile idiopathic arthritis (JIA) has revealed distinct abnormalities in function and distribution in various disease subtypes. De Kleer et al. found reduced numbers of circulating CD4+CD25^high ^regulatory T cells in extended oligoarticular JIA, compared to persistent oligoarticular JIA [106]. The numbers of CD4+CD25^high ^Foxp3+ regulatory T cells in the synovial fluid of inflamed joints were comparable, but more CD4+CD25^intermediate ^Foxp3+ regulatory T cells were present in persistent vs. extended oligoarticular JIA. Synovial fluid CD4+CD25^high ^regulatory T cells had more potent *in vitro *suppressive effects compared to their peripheral blood counterparts, suggesting possible functional enhancement of these cells in the joints. In addition, CD4+CD25^high ^regulatory T cells more easily suppress peripheral blood CD4+CD25- T effector cells than T effectors from synovial fluid, consistent with *in vitro *findings on the effects of IL-1 and IL-6 on susceptibility to suppression [107]. The authors conclude that CD4+CD25^high ^regulatory T cells cannot prevent disease development, but synovial CD4+CD25^high ^regulatory T cells may contribute to reversal of ongoing inflammation in persistent oligoarticular JIA [106,108].

In another study of synovial CD4+CD25^high ^regulatory T cells in persistent and extended oligoarticular JIA, Massa et al. demonstrated that certain epitopes of human HSP increase the frequency of CD4+CD25^high ^regulatory T cells and induce Foxp3 expression [109]. Reactivity of CD4+CD25^high ^regulatory T cells to these human HSP epitopes appears to influence regulation of inflammation in oligoarticular JIA [109].

In systemic JIA, circulating CD4+CD25^high ^regulatory T cell frequency was reported to be lower than healthy controls [110]. Studies from our laboratory showed that circulating CD4+CD25^high ^CD127^lo/- ^regulatory T cell numbers are normal, but their *in vitro *suppressive function is lower than that of healthy controls (unpublished data). This defect in CD4+CD25^high ^regulatory T cell-mediated suppression does not appear to result from a deficiency of CD45RA+ naïve cells, the more suppressive subset of CD4+CD25^high ^CD127^lo/- ^regulatory T cells (unpublished data). In contrast, we find reduced levels of circulating CD4+CD25^high ^regulatory T cells in polyarticular JIA (unpublished data).

Ruprecht et al. [111] also investigated CD4+CD25^high ^regulatory T cells in synovial fluid of patients with JIA. They found that CD4+CD25^high ^regulatory T cells expressing surface CD27 exhibit a higher level of Foxp3 and have stronger suppressive activity. They concluded that, used in conjunction with CD25, CD27 is a useful marker to distinguish regulatory from effector T cells in inflamed tissues. However, others have disputed the specificity of CD27 as a CD4+CD25^high ^regulatory T cell marker [112].

Another important issue is how various JIA treatments affect CD4+CD25^high ^regulatory T cell distribution and function. It was reported that methotrexate and corticosteroids do not influence the frequency or activity of these cells in JIA [106,110]. De Kleer et al. observed normalization of levels of circulating CD4+CD25^high ^regulatory T cell after autologous stem cell transplantation (ASCT), perhaps from the preferential homeostatic expansion of CD4+CD25^high ^regulatory T cells during the lymphopenic phase of immune reconstitution. They postulated that ASCT reprograms auto-reactive T cells and restores the immune regulatory network of CD4+CD25^high ^regulatory T cells [110].

### Rheumatoid arthritis (RA)

Reported data on frequency and activity of CD4+CD25^high ^regulatory T cells in rheumatoid arthritis (RA) are conflicting. Liu et al. found the quantities and functional properties of CD4+CD25^high ^regulatory T cells in peripheral blood of RA patients to be comparable to healthy control subjects [113,114], while Cao et al. reported a decreased frequency of CD4+CD25^high ^regulatory T cells in peripheral blood of RA subjects [114]. Some studies found that treatment with methotrexate, hydroxychloroquine, anti-TNF-α, and systemic/intra-articular steroids does not influence the abundance or suppressive function of CD4+CD25^high ^regulatory T cells [115-118], while others reported increased levels and suppressive function with TNF-α blockade [117,118].

Nevertheless, there is a consensus that synovial fluid in inflamed joints is enriched in CD4+CD25^high ^regulatory T cells [113,114,119]. These synovial CD4+CD25^high ^regulatory T cells express increased levels of inflammation-related chemokine receptors, such as CCR4, CCR5, and CXCR4 [120]. Like findings in JIA, evidence for the increased resistance of RA synovial T effector cells to suppression by CD4+CD25^high ^regulatory T cells has been reported [116]. Behrens et al. linked CD4+CD25^high ^regulatory T cell dysfunction in RA to a disturbance in the homeostatic relationship between CD4+CD25^high ^regulatory T cells and Th1 cells in the synovium. CD4+CD25^high ^regulatory T cells from RA subjects are capable of suppressing the production of IFN-γ by synovial membrane Th1 lymphocytes [121]. However, the ratio of CD4+CD25^high ^regulatory T cells to IFN-γ producing cells is lower in the synovial membrane than in synovial fluid or blood. The authors suggest that the local imbalance between Th1 and CD4+CD25^high ^regulatory T cells may be responsible for repeated rheumatic flares and could be a target for future treatments [121].

### Systemic lupus erythematosus (SLE)

Findings that central tolerance remains intact in murine models of SLE suggest a critical breakdown of peripheral tolerance in this disease [122-124]. Consistent with this possibility, most studies in human SLE indicate that CD4+CD25^high ^regulatory T cell distribution is altered in association with active disease. Numbers of circulating CD4+CD25^high ^regulatory T cells decrease in patients with active SLE [125-127] while clinical remission is associated with elevated or normal CD4+CD25^high ^regulatory T cell frequency [128-131]. A single study reported that disease activity in SLE correlates positively with the numbers of CD4+CD25^high ^regulatory T cells [131].

In a study of CD4+CD25^high ^regulatory T cell function, Vallencia et al. claimed that a reversible defect occurs in patients with SLE. CD4+CD25^high ^regulatory T cells from active but not inactive SLE patients were deficient in *in vitro *suppressive activity and had decreased Foxp3 mRNA and protein [132,133]. Opposite findings of increased Foxp3 expression in active disease were reported in one study of pediatric SLE [133]. Yan et al. found no difference in Foxp3 expression in CD4+CD25^high ^regulatory T cells of SLE patients [134]. However, decreased suppressive function of CD4+CD25^high ^regulatory T cells appeared to be a consequence of inhibition by IFN-activated autologous antigen presenting cells. These cells could also inhibit the function of CD4+CD25^high ^regulatory T cells from healthy control subjects [135].

### Other rheumatic diseases

The work on CD4+CD25^high ^regulatory T cells in other rheumatic diseases is limited to date. In primary Sjogren syndrome, Gottenberg et al. reported an increase in circulating CD4+CD25^high ^regulatory T cells, and no change in levels with methotrexate or corticosteroid treatment [136]. However, a more recent report argues that the numbers of circulating CD4+CD25^high ^regulatory T cells in patients with Sjogren syndrome decrease [137].

In Kawasaki disease, Furuno et al. found that during the active phase of the disease, the number of circulating CD4+CD25^high ^regulatory T cells is reduced compared to patients with infectious causes of febrile illness, whose CD4+CD25^high ^regulatory T cell numbers are higher than in healthy subjects. In defervesce phase of the disease, the number of CD4+CD25^high ^regulatory T cells in patients with Kawasaki disease increases to/or above normal levels, while CD4+CD25^high ^regulatory T cells in patients with infectious febrile disease decrease to normal levels [138].

In spondyloarthropathy, a single study by Cao et al. found normal levels of circulating CD4+CD25^high ^regulatory T cells, but a higher proportion of CD4+CD25^high ^regulatory T cells in synovial fluid of inflamed joints than in peripheral blood [114].

In sarcoidosis, Miyara et al. showed an increase in frequency of CD4+CD25^high ^regulatory T cells in sarcoid granulomas, bronchoalveolar lavage fluid (BALF), and peripheral blood of patients with active disease. The cells reportedly exhibit powerful anti-proliferative activity, but cannot completely inhibit TNF-α production. The authors conclude that although sarcoidosis is associated with global CD4+CD25^high ^regulatory T cell amplification, the cells are functionally insufficient to control local inflammation [139]. In contrast, Idali et al. [140] found decreased frequency of Foxp3+ cells among BALF and blood CD4+ cells in sarcoidosis patients.

### Mechanistic issues

Current data indicate that reduced numbers of circulating CD4+CD25^high ^regulatory T cells is not a general finding in rheumatic diseases, while reduced function is more commonly found. Several hypothetical defects in CD4+CD25^high ^regulatory T cell function that could lead to autoimmunity have been proposed [141]. However, data pointing to a secondary effect on CD4+CD25^high ^regulatory T cells in autoimmune disorders have also emerged. The example of SLE is illustrative. Compromised function could result from direct interaction between SLE-associated auto-antigens and their cognate ligands on CD4+CD25^high ^regulatory T cells [142]. Alternatively, endogenous stimulants in SLE may activate antigen presenting cells to produce alpha-interferon and related factors that inhibit CD4+CD25^high ^regulatory T cell activity [134]. Pro-inflammatory factors associated with autoimmunity, such as IL-1, IL-6, and TNF-α, also can inhibit CD4+CD25^high ^regulatory T cell function [143-145]. The resolution of this issue is central to a full understanding of autoimmunity.

Increased suppressive potency of CD4+CD25^high ^regulatory T cells at sites of inflammation has been reported in several diseases. The relative importance of circulating versus tissue CD4+CD25^high ^regulatory T cells requires more study. One attractive possibility is that tissue CD4+CD25^high ^regulatory T cells may be more antigen-specific, and consequentially more suppressive [106,116] while circulating CD4+CD25^high ^regulatory T cells may be recruited to different tissues in response to inflammatory conditions [146], and non-specifically augment suppression. The occasionally reported reduction in numbers of CD4+CD25^high ^regulatory T cells in the circulation may result from their recruitment to sites of inflammation. However, expansion of tissue localized and circulating CD4+CD25^high ^regulatory T cells may occur during autoimmune-associated inflammation [116]. Thus, CD4+CD25^high ^regulatory T cells may be actively recruited or be generated de novo at sites of inflammation (or both). It is anticipated that the development of new technologies that allow in vivo tracking of circulating CD4+CD25^high ^regulatory T cells will advance our current understanding of migratory and suppressive potentials of different subsets of CD4+CD25^high ^regulatory T cells. Finally, the potent suppressive activity of CD4+CD25^high ^regulatory T cells at inflammatory sites is usually insufficient to control inflammation. One probable explanation is that the presence of inflammatory cytokines at these sites makes effector T cells more resistant to suppression. In addition, the recently reported induction of highly inflammatory Th17 cells from CD4+CD25^high ^regulatory T cells that are not terminally differentiated [147] suggests that the latter may, under certain conditions, potentiate rather than suppress inflammation.

### CD4+CD25^high ^regulatory T cells as a treatment in autoimmune and rheumatic diseases

There is a need to carefully control the size of the CD4+CD25^high ^regulatory T cell population *in vivo *to achieve a balance between the necessity to suppress auto-reactivity and the ability to allow appropriate responses to foreign and tumor antigens. Little is known of the mechanisms of this control; however, the alterations in distribution and function of CD4+CD25^high ^regulatory T cells in autoimmune and rheumatic diseases suggest a role for the therapeutic use of these cells. In mice with collage-induced arthritis, depletion of CD4+CD25^high ^regulatory T cells causes rapid progression, and the transfer of isolated and *ex vivo*-proliferated CD4+CD25^high ^regulatory T cells can reverse early joint damage [148]. Administration of CD4+CD25^high ^regulatory T cell also yields improvement in murine models of colitis, autoimmune encephalomyelitis, diabetes, and allogeneic transplantion [149-152].

Human research has shown that some established therapies may promote CD4+CD25^high ^regulatory T cell development and survival *in vivo*. For instance, monoclonal antibody to CD20 (rituximab), which depletes B cells, leads to a selective increase in CD4+CD25^high ^regulatory T cells [153]. Polyclonal antibody therapies, such as anti-lymphocyte serum (ALS) and anti-thymocyte globulin (ATG), have been shown to preferentially deplete T effector cells, and induce CD4+CD25^high ^regulatory T cells [154,155]. As described above, rapamycin preferentially expands CD4+CD25^high ^regulatory T cells. Therefore, a major therapeutic effect of rapamycin may be the induction of tolerogenic CD4+CD25^high ^regulatory T cells *in vivo*.

Besides these established therapies, recent research has focused on cytokine related therapies to modulate CD4+CD25^high ^regulatory T cell function. Among candidate cytokines are growth factors in the IL-2 family. These cytokines signal via STAT5, the homeostatic pathway that regulates CD4+CD25^high ^regulatory T cell function. Several studies have reported that these cytokines enhance immune regulation by CD4+CD25^high ^regulatory T cells. For instance, IL-7 and IL-15 are involved in the preservation of optimal suppressive function by CD4+CD25^high ^regulatory T cells [156]. In addition, IL-15 administration alone induces *de novo *generation of CD4+CD25^high ^regulatory T cells [157]. The newly identified IL-35 has been shown to trigger CD4+CD25^high ^regulatory T cell expansion and subsequent immune suppression [158]. However, the specificity of these cytokines for CD4+CD25^high ^regulatory T cells needs to be further examined to avoid undesirable expansion of effector T cells.

In contrast to T cell growth factors, pro-inflammatory cytokines have been shown to inhibit function of CD4+CD25^high ^regulatory T cells, possibly via promotion of Th17 development [159]. Therefore, anti-TNF-α, anti-IL1, anti-IL6, and anti-IL-21 therapies may affect inflammation not only by direct inhibition of the pro-inflammatory cytokines but also by reestablishment of immune regulation by CD4+CD25^high ^regulatory T cells. On the other hand, short term treatment with high dose CTLA-4Ig (abatacept), which has been shown to have anti-inflammatory properties in arthritis, leads to a precipitous loss of CD4+CD25^high ^regulatory T cells and, in some animal models, exacerbation of autoimmunity [160].

Direct transfusion of CD4+CD25^high ^regulatory T cell in humans is starting to be explored as a therapy. We are aware of two early trials in patients post stem cell transplantation (SCT). In patients with allogeneic SCT, Matthias Edinger and his team from the Department of Hematology and Oncology at the University Hospital of Regensburg, Germany are conducting a phase I clinical trial (safety and feasibility) using CD4+CD25^high ^regulatory T cells-enriched lymphocyte products (personal communication). Patients with a high risk of relapse after allogeneic SCT are preemptively treated with donor T cells enriched with 50–60% of CD4+CD25^high ^regulatory T cells, in order to reduce GVHD. Eight patients have been treated so far without complications. A trial using third party cord blood CD4+CD25^high ^regulatory T cell in patients with SCT has been recently initiated at the University of Minnesota (Dr. B. Balazar, personal communication). We are not aware of any established clinical trials in autoimmune diseases, although CD4+CD25^high ^regulatory T cell therapy will possibly be initiated in type 1 diabetes in the near future.

Despite encouraging data from animal models and early human trials, a number of issues must be resolved for optimal use of CD4+CD25^high ^regulatory T cells as a therapy [161,162]. Firstly, there are likely to be differences in the specific role of CD4+CD25^high ^regulatory T cells in particular diseases, and these must be elucidated. Secondly, CD4+CD25^high ^regulatory T cell-specific surface markers remain elusive, which hampers the isolation of pure populations of CD4+CD25^high ^regulatory T cells. Third, the use of autologous CD4+CD25^high ^regulatory T cell clones for particular auto-antigens would increase the effectiveness and decrease potential side effects of "bystander" suppression. This will require techniques for identifying and expanding antigen specific clones of CD4+CD25^high ^regulatory T cells. Recent successes with CD4+CD25^high ^regulatory T cell expansion using rapamycin are promising in this regard [163,164]. Lastly, the fate of transfused CD4+CD25^high ^regulatory T cells *in vivo *is not fully known. In the unlikely event that CD4+CD25^high ^regulatory T cells expand into tumor/effector cells or simply become broadly immunosuppressive, there needs to be a way to eliminate them from the body. Future therapies may require the use of "designer" CD4+CD25^high ^regulatory T cells that have been modified by gene transfer to selectively express preferred proteins including antigen specific TCR, homing receptors, cytokines, and "suicide" genes [161,162]. Nevertheless, the manipulation of CD4+CD25^high ^regulatory T cell function shows great promise as a novel therapeutic option in autoimmune and rheumatic diseases.

## Competing interests

The authors declare that they have no competing interests.

## Authors' contributions

DM has formulated the concept and design of the manuscript and has written the review. KDN critically revised the initial manuscript and created the figures. DW has been involved in revising the manuscript. EDM has made critical contributions to the concept, design, and revision of the manuscript. All authors read and approved the final manuscript.
